# Supplementary material for: RSG-YOLOV8: Detection of rice seed germination rate based on enhanced YOLOv8 and multi-scale attention feature fusion
Source: PLoS One. 2024 Nov 12;19(11):e0306436. doi: 10.1371/journal.pone.0306436 (PMC11556723; doi:10.1371/journal.pone.0306436)
Supplement: Attachment — Submitted filename: Revising Report.docx [file pone.0306436.s001.docx]

Dear Editor and Reviewers:

Thank you for your letter and for the reviewers’ comments concerning our manuscript entitled “**RSG-YOLO: Detection of rice seed germination rate based on enhanced YOLOv8 and multi-scale attention feature fusion (Manuscript ID: PONE-D-24-24638)**”. These comments are very helpful for revising and improving our paper, as well as the important guiding significance to our research. We have studied these comments carefully and have made correction. The main corrections in the paper and the responds to the reviewer’s comments are as following:

**Responses to Reviewer #1**

Reviewer #1: The paper presents a novel RSG-YOLO model with promising enhancements. Please clarify the below points to strengthen the manuscript's contribution

1.Can you provide more details on the Rice Seed Germination dataset used for validation? How was the dataset curated to ensure its representativeness and reliability for training and evaluating the RSG-YOLO model?

***Reply*：**Thank you for your interest in the research and your valuable questions. Regarding the validation details of the rice seed germination dataset you inquired about, additional information is provided here.

To ensure the representativeness and reliability of the dataset in training and evaluating the RSG-YOLO model, the following measures were taken: first, the inclusion of rice seed images from different populations in the dataset ensured the diversity of the samples. This diversity helps the model to learn the germination characteristics under different conditions, thus improving its representativeness and reliability. Second, when dividing the training and test sets, a random sampling approach is adopted to avoid potential bias. This approach ensures the representativeness of the training and test data, allowing the model to be effectively evaluated on unseen data. Finally, during the training process, a number of data enhancement techniques are also applied to increase the diversity of the training samples. These techniques include rotation, scaling and flipping, etc., which aim to improve the robustness and accuracy of the model. It is believed that through the above measures, the dataset can effectively support the training and evaluation of the RSG-YOLO model and ensure the reliability and validity of the results.

**In 2.1 Dataset section**

The rice seed germination dataset used in this study is the publicly available RiceSeedGermination dataset from the official Kaggle webpage. The dataset contains images of rice seeds from nine different populations, demonstrating a wide range of phenotypic diversity between varieties, including variations in length, shape and color. Table 1 lists some of the grain seeds with different phenotypic characteristics that vary considerably in length, shape, and color. In total, there are 600 rice seed images, which are divided into a training set and a test set with a ratio of 8:2.

Table 1. The grain seeds with different phenotypic characteristics

| Image | Grain length | Grain shape | Grain color | Awn |
| --- | --- | --- | --- | --- |
|  | Short | Bold | Red | Yes |
|  | Short | Bold | Dark brown | Yes |
|  | Medium | Slender | Light brown | Yes |
|  | Short | Bold | Yellow | No |
|  | Short | Bold | Red | No |
|  | Medium | Medium | Yellow | No |
|  | Short | Bold | Yellow | No |

2.The BRA module is described as a dynamic and sparse attention mechanism. Can you provide quantitative details on how BRA compares to other attention mechanisms (e.g., SE, CBAM, ECA) in terms of performance improvements and computational overhead? How does BRA’s dynamic and sparse nature contribute to the reduction of redundancy and enhancement of critical feature highlighting?

***Reply***: Thank you for your valuable feedback. Regarding the comparison of the BRA module's performance improvement and computational overhead with other attention mechanisms (e.g., SE, CBAM, ECA), the following quantitative details will be provided.

Regarding computational overhead, the computational complexity of BRA is$O((HW)^{(\frac{4}{3})})$, while that of the conventional global self-attention mechanism is $O((HW)^{2})$, which suggests that BRA has a lower computational overhead when processing high-resolution inputs. In addition, BRA is able to perform key-value pair selection at a coarser region level through region-level graph construction and pruning, which reduces redundant computation. This design allows BRA to effectively reduce the consumption of computational resources while maintaining high performance.

The dynamic nature of BRA allows it to adjust the attention distribution based on variations in input features, thus enhancing detection capabilities by highlighting key features under different conditions. Its sparse characteristics further optimize feature selection by eliminating unnecessary key-value pairs, which reduces processing of redundant information and improves both efficiency and accuracy.

**In 1.2.2 BRA-Based Attention Feature Fusion and 3.2.3 Comparison of different attention mechanisms section**

the BRA module demonstrates a clear performance improvement over other attention mechanisms, such as SE, CBAM, and ECA. In the RSG-YOLO model, BRA achieves an mAP_50_ of 0.981, which notably surpasses CBAM's 0.976 and ECA's 0.944. This superior performance highlights BRA's effectiveness in enhancing detection accuracy.

Table 2. Ablation experiments of attention mechanisms. Replace BRA in RSG-YOLOV8 with SE, ECA, CBAM, CA, RFA respectively. The best results are highlighted in bold.

| Model | Precision | Recall | F1-score | mAP_50_ | mAP_50-95_ |
| --- | --- | --- | --- | --- | --- |
| SRSG-YOLOV8 | 0.918 | 0.864 | 0.890 | 0.954 | 0.650 |
| ERSG-YOLOV8 | 0.942 | 0.875 | 0.907 | 0.944 | 0.782 |
| CRSG-YOLOV8 | **0.959** | 0.856 | 0.905 | 0.976 | 0.672 |
| ARSG-YOLOV8 | 0.936 | 0.873 | 0.903 | 0.958 | 0.752 |
| RRSG-YOLOV8 | 0.905 | 0.928 | 0.916 | 0.945 | 0.772 |
| **RSG-YOLOV8** | 0.942 | **0.968** | **0.955** | **0.981** | **0.779** |

3.How does the GFPN-based structured feature fusion network improve feature integration compared to the original Neck component of YOLOv8? Please detail the advantages of GFPN’s approach in handling multi-scale features and its impact on overall model performance.

***Reply***: Thank you for your insightful question regarding the GFPN-based structured feature fusion network and its advantages over the original Neck component of YOLOv8. The improvements GFPN brings to feature integration can be highlighted in several aspects.

**In 1.2.3 GFPN section**

Firstly, GFPN significantly enhances the flow of information between different layers through the use of dense connections and the Queen-Fusion structure.

As shown in Fig. 5, the Queen-fusion connection in P_5_ consists of the previous layer P_4_ downsampling, the previous layer P_6_ upsampling, the previous layer P_5_ and the current layer P_4_. In this work, bilinear interpolation and maximum pooling are applied as the upsampling and downsampling functions, respectively. Therefore, in the case of extreme large-scale changes, the model is required to have sufficient exchange of information between the upper and lower layers. Based on the mechanism of layer-hopping and cross-scale connectivity, GFPN can be as long as the ‘giraffe's neck’. With such a ‘heavy neck’ and a lightweight backbone, RSG-YOLOV8 can strike a balance between higher accuracy and better efficiency.

This design allows for a more comprehensive integration of features, as it retains all relevant information from various layers without the risk of losing critical details, which can occur in traditional feature fusion methods. This enhanced feature integration capability enables the model to more accurately identify objects in complex scenarios.

Secondly, GFPN is specifically designed to address the challenges associated with multi-scale feature integration. By employing a top-down approach to feature fusion, GFPN effectively combines high-level semantic features with low-level spatial details. This is crucial for object detection tasks, where objects can vary significantly in size. GFPN facilitates the extraction of features across different scales, ensuring that the model can accurately detect small, medium, and large objects.

Additionally, GFPN minimizes the risk of information loss during the fusion process by using the Concat operation instead of traditional summation methods. By concatenating features from different layers, GFPN ensures that all relevant information is preserved, leading to richer feature representations that enhance the model's ability to make accurate predictions.

Finally, the implementation of GFPN in the RSG-YOLO model has shown substantial performance improvements. Experimental results indicate that RSG-YOLO achieves an mAP_50_ of 0.981, representing a 4% improvement over the original YOLOv8 model. This enhancement is attributed to the effective multi-scale feature fusion facilitated by GFPN, which allows the model to better discern crucial details within the input data.

Figure. 5. Illustration of Queen-fusion in GFPN. C represent concatenation fusion style, and ’P_k_ denotes node in next layer

4.The introduction of an additional detection head is mentioned. What specific improvements in detection performance (e.g.,precision, recall) were observed with this modification? How do variable anchor box scales and optimized regression losses contribute to these performance improvements?

***Reply***: Thank you very much for your comments. The introduction of additional detection heads into the RSG-YOLO model resulted in a significant improvement in the detection performance metrics, especially in terms of precision and recall.

The experimental results show that the RSG-YOLO model achieves an average precision (mAP50) of 0.981, which is 4% higher than the original YOLOv8 model. In addition, the recall of the RSG-YOLO model is 0.968, which is 7.1% higher than the baseline YOLOv8 model without enhancements.

**In 1.2.4 Added Detection Head section**

The specific improvement in detection performance can be attributed to two key factors: firstly, the implementation of variable anchor frame scales allows the model to better match the size of objects in the input image. By using multiple scales, the detection head can more accurately localize and classify objects of different sizes, which is particularly important in tasks such as rice seed detection, where the size of objects can vary greatly. Secondly, optimization of regression loss within the detection head plays a crucial role in improving model performance. By improving the way the model calculates the loss associated with bounding box predictions, the model can learn from errors more efficiently, leading to better localization of detected objects and ultimately improved precision and recall.
5.What specific steps were taken to ensure that RSG-YOLO performs robustly under varying conditions such as different lighting scenarios or camera angles? How does the model's performance vary across these conditions?

***Reply***: Thank you for your interest in the research and your valuable questions. the importance of evaluating model performance under different lighting conditions and camera angles is fully recognized.

In this study, due to the limitations of the dataset, all images were captured under identical lighting conditions and camera angles. Consequently, the current experimental results predominantly reflect the performance of the RSG-YOLO model under these specific conditions. It is acknowledged that future research could further validate the model's robustness by introducing diverse lighting and shooting angles.

Nevertheless, the superior performance of the RSG-YOLO model under these particular circumstances establishes a foundation for its potential in broader applications. There is an anticipation of exploring the model's performance under varying conditions in subsequent work to enhance its practicality further.

6.Can you elaborate on how the integration of CSP DenseNet and the BRA attention mechanism interacts with the YOLOv8 architecture to enhance detection performance? How do these architectural changes affect the trade-offs between computational complexity and accuracy?

***Reply***: Thank you for your in-depth questions. An elaboration on how CSP DenseNet and the BRA attention mechanism interact with the YOLOv8 architecture to enhance detection performance would be a pleasure.

**In 1.2.1 CSPDenseNet and 1.2.2 BRA-Based Attention Feature Fusion section**

First, CSP DenseNet significantly improves feature transfer and reuse capabilities by introducing a design that is partially connected across stages. Building on YOLOv8, the integration of CSP DenseNet allows information to flow more efficiently between the different layers of the network, thus enhancing feature representation. This densely connected structure not only helps to capture more detailed information, but also mitigates the problem of gradient vanishing, thus speeding up the convergence of the model.

Secondly, the introduction of the BRA attention mechanism further optimizes the feature extraction process. BRA is able to highlight important features and reduce redundant information through a dynamic and sparse attention mechanism. This mechanism ensures that the model is able to focus on the most relevant features when processing the input data, which improves the accuracy of the detection. Combined with the feature delivery capability of CSP DenseNet, the BRA mechanism makes the model more efficient in multi-scale feature fusion, further improving the detection performance of YOLOv8 in complex scenarios.

Regarding the trade-off between computational complexity and accuracy, although the integration of CSP DenseNet and the BRA mechanism increases the complexity of the model to a certain extent, this issue is addressed by optimizing the network architecture and incorporating efficient feature fusion strategies, such as GFPN. The experiments show that despite the increase in the number of parameters of the model, this increase is acceptable compared to the significant improvement in performance. In addition, when the camera angle deviates significantly from the training scene, the model may experience difficulties in object localization, especially when the shape of the object is distorted. It is acknowledged that these factors may influence the overall performance of the model. Thus, ongoing evaluation and fine-tuning will be undertaken to further enhance its performance under various anticipated conditions.

**Responses to Reviewer #2**

Reviewer #2: The manuscript can be published with the following changes:

1. The version of yolo modified can be mentioned in proposed method name also.

***Reply***: Thank you for your comment. The specific version of YOLO utilized in the method's designation has been incorporated to elucidate the proposed approach more distinctly. The new name is ‘RSG-YOLOv8’ and has been updated in the relevant parts of the paper to ensure consistency.

2. Block diagram of the proposed system needs to be added.

***Reply***: Thank you for your valuable comments. A diagram of the overall structure of the RSG-YOLOv8 model has been added prior to receiving revisions (see Figure 2). The diagram shows in detail the components of the model and their interrelationships so that the reader can better understand the design of the system.

3. why F1 score is not used for evaluation justify.

***Reply***: Thank you for your valuable comments. The significance of the F1 score as an assessment metric is duly acknowledged, and a pertinent discussion of this metric has been incorporated into the revised article.

In the study, the F1 score serves as a complementary metric for evaluating model performance, thereby offering a more comprehensive perspective on performance across various categories. A detailed explanation of how the F1 score is calculated and its role in the evaluation process is provided in the 3.1 Performance Indicators section of the paper.

Figure. 7. Change curves of evaluation metrics of YOLOv5, YOLOv7, YOLOv8, YOLO-r, and RSG-YOLOV8 models

Figure 7(e) shows the dynamics of F1-score over epochs. YOLOv5's F1-score rises smoothly with increasing epochs, demonstrating a stable training process; YOLOv7 is similar to YOLOv5, but has a faster performance improvement rate, reaching higher F1-score at certain epoch points; RSG-YOLOV8 does not have a high F1-score at the beginning, but with increasing epochs, the F1-score increases significantly, eventually surpassing other YOLO series models; YOLOv8 maintains high F1-score throughout the training process, showing good stability and generalisation ability; YOLO-r has a more fluctuating trend in F1-score, but also improves with epochs increase in general, but not as significantly as the other models.

4. The research gap leading to methodology needs to be mentioned at the end of literature review.

***Reply***: Thank you for your valuable comments. A discussion of the research gaps has been included in the concluding section of the literature review.

**In Introduction section:**

A comprehensive analysis of the existing literature reveals that although significant progress has been made in the field of target detection, there are still some research gaps. Firstly, the effectiveness of existing models in dealing with multi-scale feature fusion is still limited, especially in the task of target detection in complex contexts. In addition, although multiple attention mechanisms have been proposed, their performance differences in different application scenarios have not been fully explored.

5. The following references can be added

i) Venkatesan, R., & Balaji, G. N. (2024). Balancing composite motion optimization using R-ERNN with plant disease. Applied Soft Computing, 154, 111288

ii) Appe, S. N., Arulselvi, G., & Balaji, G. N. (2023). Detection and Classification of Dense Tomato Fruits by Integrating Coordinate Attention Mechanism With YOLO Model. In Handbook of Research on Deep Learning Techniques for Cloud-Based Industrial IoT (pp. 278-289). IGI Global.

***Reply***: Gratitude is extended for the insightful comments. Upon reviewing these papers, the references have been incorporated into the paper and are references 10 and 11.
